# Association between the Cardiac Arrest Hospital Prognosis (CAHP) score and reason for death after successfully resuscitated cardiac arrest

**DOI:** 10.1038/s41598-023-33129-8

**Published:** 2023-04-13

**Authors:** Marine Paul, Stéphane Legriel, Sarah Benghanem, Sofia Abbad, Alexis Ferré, Guillaume Lacave, Olivier Richard, Florence Dumas, Alain Cariou

**Affiliations:** 1 Intensive Care Unit, Centre Hospitalier de Versailles-Site André Mignot, 177 Rue de Versailles, 78150 Le Chesnay, France; 2 AfterROSC Study Group, Paris, France; 3 University Paris-Saclay, UVSQ, INSERM, CESP, Team “PsyDev”, Villejuif, France; 4 SAMU 78, Centre Hospitalier de Versailles-Site André Mignot, Le Chesnay Cedex, France; 5 Intensive Care Unit, Cochin Hospital (APHP), Paris, France; 6 Sorbonne Paris Cité-Medical School, Paris Descartes University, Paris, France; 7 Paris-Cardiovascular-Research-Center, INSERM U970, Paris, France; 8 Paris Sudden Death Expertise Centre, Paris, France; 9 Emergency Department, Cochin Hospital, Paris, France

**Keywords:** Epidemiology, Outcomes research, Clinical trial design

## Abstract

Individualize treatment after cardiac arrest could potentiate future clinical trials selecting patients most likely to benefit from interventions. We assessed the Cardiac Arrest Hospital Prognosis (CAHP) score for predicting reason for death to improve patient selection. Consecutive patients in two cardiac arrest databases were studied between 2007 and 2017. Reasons for death were categorised as refractory post-resuscitation shock (RPRS), hypoxic-ischaemic brain injury (HIBI) and other. We computed the CAHP score, which relies on age, location at OHCA, initial cardiac rhythm, no-flow and low-flow times, arterial pH, and epinephrine dose. We performed survival analyses using the Kaplan–Meier failure function and competing-risks regression. Of 1543 included patients, 987 (64%) died in the ICU, 447 (45%) from HIBI, 291 (30%) from RPRS, and 247 (25%) from other reasons. The proportion of deaths from RPRS increased with CAHP score deciles; the sub-hazard ratio for the tenth decile was 30.8 (9.8–96.5; *p* < 0.0001). The sub-hazard ratio of the CAHP score for predicting death from HIBI was below 5. Higher CAHP score values were associated with a higher proportion of deaths due to RPRS. This score may help to constitute uniform patient populations likely to benefit from interventions assessed in future randomised controlled trials.

## Introduction

Mortality after cardiac arrest (CA) is very high and chiefly due to refractory post-resuscitation shock (RPRS), hypoxic-ischaemic brain injury (HIBI), and brain death^1^. Preventing HIBI is considered crucial, since withdrawal of life-sustaining treatments (WLST) warranted by a poor neurological prognosis is the most common reason for death^2^. Despite decades of research, few treatments have been shown to improve outcomes after CA^3–8^. Targeted temperature management (TTM) has long been considered the only effective neuroprotective treatment. In the HYPERION randomised trial, TTM at 33 °C improved outcomes in patients with an initial non-shockable rhythm compared to normothermia^9^. However, TTM failed to provide benefits in other studies, including the TTM2 randomised controlled trial, which also compared 33 °C to normothermia^8^. One possibility is that specific treatments may benefit some patient sub-groups but not others, and that trials have included patients with heterogeneous phenotypes associated with different reasons for death. For instance, in the TTM2 trial^8^, a substantial proportion of patients died from RPRS with multiorgan failure within 48 h after intensive-care-unit (ICU) admission. Such patients are unlikely to benefit from neuroprotective interventions, and their inclusion in the trial is therefore criticisable^10^. The ability to predict the most likely reason for death might improve patient selection for specific treatment strategies.

We hypothesised that the severity of illness at ICU admission might be associated with the reason for death in the ICU. The Cardiac Arrest Hospital Prognosis (CAHP) score has been proven effective in predicting the risk of death based on seven variables readily available at ICU admission (age, CA in a public place vs. at home, initial rhythm, no-flow and low-flow times, admission pH, and total epinephrine dose), with higher scores being associated with worse outcomes^11–13^. The objective of this study was to assess whether the CAHP score at ICU admission also predicted the reason for death, notably RPRS and HIBI.

## Methods

We used prospectively established databases from two tertiary referral centres for CA in the Paris area, France (#NCT03594318). Data collection was approved by the Ethics Committee of the French Intensive Care Society [#CESRLF_12-384 (November, 14, 2012) and 20–41 (May, 5, 2020)] which waived the requirement for written consent in accordance with French law on retrospective studies of anonymized data. The study was conducted according to French health authorities’ regulations (French Data Protection Authority #MR004_2209691. All procedures involving the patients complied with the ethical standards of the institutional and national research committees and with the 1964 Declaration of Helsinki and its later amendments. The study is reported according to the STROBE statement.

### Study population and objectives

Inclusion criteria were non-traumatic out-of-hospital cardiac arrest (OHCA) between 2007 and 2017, ICU admission with sustained return of spontaneous circulation (ROSC), age older than 18 years, and availability of the data needed to compute the CAHP score.

The primary study objective was to assess potential associations linking the CAHP score to the reason for death after OHCA. The secondary objectives were to determine levels of CAHP scores associated with death from RPRS and from HIBI.

### Post-resuscitation care

The management protocol for patients admitted to ICU after OHCA has been described elsewhere^14–16^. The only changes during the study period involved the sedation and neuromuscular blockade protocols (Electronic Supplementary Material [ESM]). In the event of persistent coma 72 h after the ROSC, sedation was discontinued and multimodal neuroprognostication carried out (ESM)^17–19^. In patients with preserved N20 peaks, preserved cranial reflexes, and a motor Glasgow Coma Scale score above 2, life-sustaining treatments were continued to allow investigations for a confounding factor such as sepsis, residual sedation, or a concomitant disease. A WLST decision was made collegially if no treatable factor was identified.

### Data collection

In each of the two prospective databases, the Utstein style^20^ was followed to collect demographic data and data related to the OHCA including age, sex, location at OHCA, first recorded rhythm, no-flow time, low-flow time, presence of a witness, cardiopulmonary resuscitation by a bystander, number of defibrillations, and epinephrine administration. The following variables were also collected in the ICU: use of TTM, development of post-resuscitation shock, admission blood lactate, and cause of OHCA. The CAHP score was computed for each patient.

To investigate whether the CAHP score predicted the reason for death, we retrospectively reviewed the ICU records to determine the reason for death categorised as RPRS, HIBI, or other (e.g., brain death; recurrent CA and WLST warranted by comorbidities) (ESM). Death from RPRS was defined as progressive, refractory haemodynamic failure despite aggressive critical care, with or without WLST. Death from HIBI was defined as WLST warranted by the results of multimodal neuroprognostication indicating a very low likelihood of neurological recovery^1^. Classification of death reason was performed by 2 authors (MP and SB), blinded from each other and a third (SA) in case of disagreement.

### Statistical analysis

Quantitative parameters were described as median [interquartile range] and qualitative parameters as proportion (percentage). For comparisons between categorical variables, we applied Pearson’s or Fisher’s test, as appropriate.

We divided the CAHP scores into deciles, instead of the usual 3 grades, to potentiate CAHP discrimination for prediction of mode of death. For each decile, we determined the outcome with the distribution of the three reasons for death (RPRS, HIBI, other). We plotted cumulative incidence curves for each reason for death over time and in terms of CAHP decile and compared them using the Fine-and-Gray method^21,22^. We then built competing-risks regression models for reasons for death in the ICU, with computation of the sub-hazard ratio and of its 95% confidence interval (95% CI) for each CAHP score decile. A sensitive analysis was performed, restricted to cardiac cause of cardiac arrest and a second restricted to initial shockable rhythm.

All tests were two-sided, with *p* values below 0.05 considered statistically significant. The frequency of missing data was less than 5% and we therefore conducted a complete-case analysis. For the statistical analyses, we used STATA/SE 14.0 (College Station, TX, USA).

## Results

**Figure 1:**
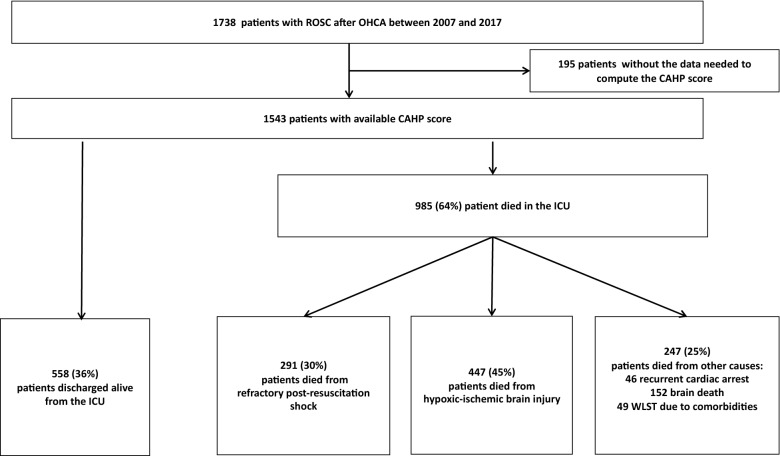
Flow diagram. *CAHP* cardiac arrest hospital prognosis, *ICU* intensive care unit, *WLST* withdrawal of life-sustaining treatments.

Figure 1 is the patient flow chart. Among the 1543 included patients, 985 (64%) died during the ICU stay, including 291 (30%) who died of RPRS and 447 (45%) of HIBI. Table 1 reports the main patient characteristics.

**Table 1:** Main characteristics in 1543 included patients with sustained return of spontaneous circulation after cardiac arrest.

N (%) or median (interquartile range)	
Patients characteristics	Total n = 1543
Age, years, median [IQR]	63 [52.4–74.1]
Males, n (%)	1084 (70)
Ischemic heart disease, n (%)	347 (22)
Kidney insufficiency, n (%)	108 (7)
Arterial hypertension, n (%)	730 (47)
Previous stroke, n (%)	90 (6)
Peripheral arterial obstructive disease, n (%)	96 (6)
Diabetes mellitus, n (%)	279 (18)
In public place at OHCA, n (%)	574 (37)
Cardiac arrest witnessed, n (%)	1393 (90)
Bystander CPR, n (%)	788 (60)
Initial shockable rhythm, n (%)	761 (49)
Number of defibrillations before ROSC, median [IQR]	1 [0–2]
Use of adrenaline, n (%)	1030 (67)
Total adrenaline dose before ROSC, mg, median [IQR]	2 [0–4]
No-flow time^a^, min, median [IQR]	3 [0–8]
Low-flow time^b^, min	19 [10–28]
Blood lactate at ICU admission, mmol/L, median [IQR]	4.7 [2.4–8.9]
CAHP score^c^, n (%)	
< 150	521 (34)
150–200	539 (35)
> 200	483 (31)
Cause of OHCA, n (%)	
Cardiac	857 (56)
Respiratory	384 (25)
Neurologic	84 (6)
Metabolic	33 (2)
Other	97 (6)
Undetermined	88 (6)
TTM (32–36 °C) on the first ICU day, n (%)	1394 (90)
Post-resuscitation shock, n (%)	1142 (74)

*IQR* interquartile range, *OHCA* out-of-hospital cardiac arrest, *CPR* cardiopulmonary resuscitation, *ROSC* return of spontaneous circulation, *CAHP* Cardiac Arrest Hospital Prognosis, *TTM* targeted temperature management.

^a^No-flow time was defined as the time from collapse to cardiopulmonary resuscitation initiation.

^b^Low-flow time was defined as the time from cardiopulmonary resuscitation initiation to the return of spontaneous circulation.

^c^CAHP scores are divided into three risk categories: < 150, low risk; 150–200, moderate risk; and > 200, high risk of poor outcomes.

### Outcomes in each CAHP score decile

**Figure 2:**
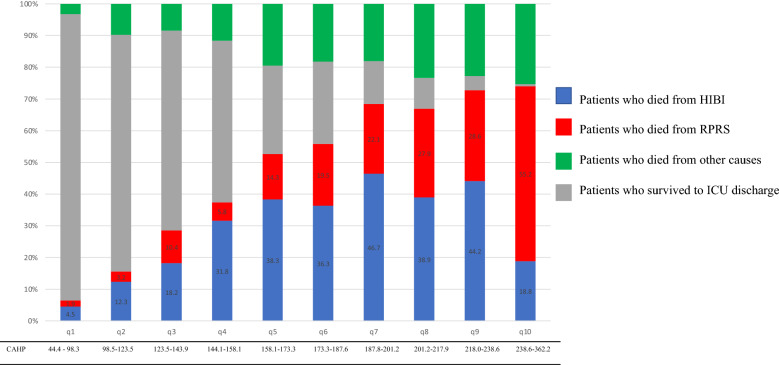
Outcomes according to CAHP score decile. The distribution of outcome is in percent. Each decile of CAHP had 154 patients. *CAHP* cardiac arrest hospital prognosis, *HIBI* hypoxaemic–ischaemic brain injury, *RPRS* refractory post-resuscitation shock, *ICU* intensive care unit.

CAHP scores ranged from 44.4 to 362.2. Figure 2 shows patient outcomes at ICU discharge in each CAHP decile. Higher CAHP scores were significantly associated with worse outcomes, with a 99% mortality rate in the tenth decile (*p* < 0.0001).

Starting at the fifth CAHP decile, the proportion of deaths due to RPRS increased from one decile to the next. In the tenth decile, 55.2% of patients died of RPRS and 18.9% of HIBI.

**Figure 3:**
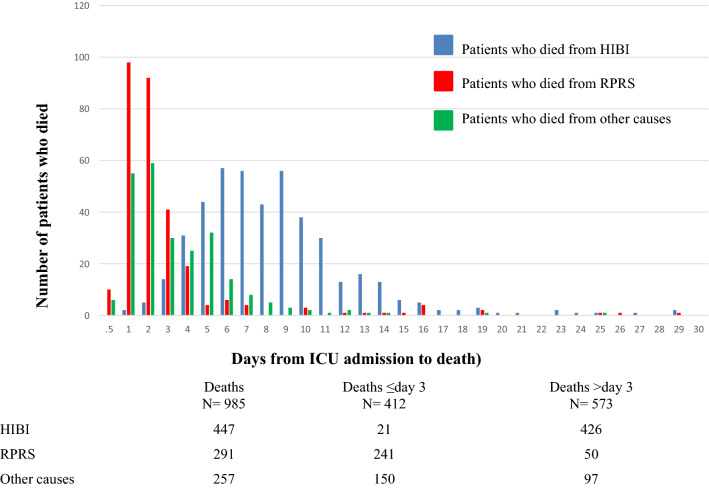
Reasons for death after out-of-hospital cardiac arrest over time in the intensive care unit. *HIBI* hypoxic-ischaemic brain injury, *RPRS* refractory post-resuscitation shock, *ICU* intensive care unit.

Median time to death in the overall population was 5 days [2–8 days] (Fig. 3). The cause of death differed according to time since ICU admission. Thus, of the 412 patients who died during the first 3 days, 241 (58%) died of RPRS. In contrast, of the 573 patients who died on day 4 or later, 426 (74%) died of HIBI.

**Figure 4:**
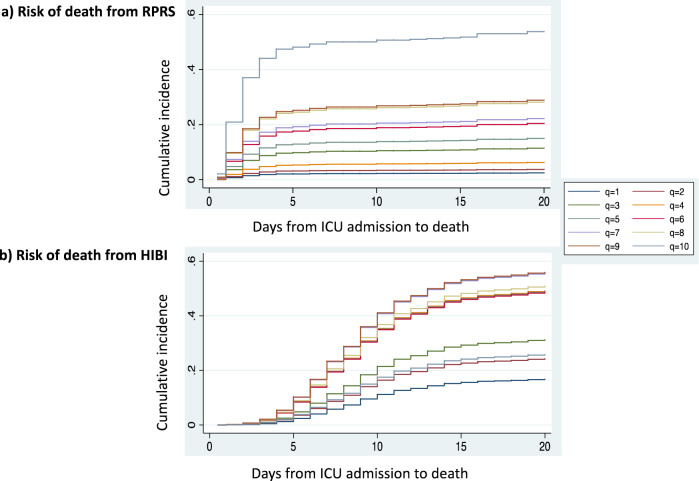
Cumulative incidence of each reason for death overtime in each CAHP score decile. (**a**) Risk of death from RPRS. (**b**) Risk of death from HIBI. *CAHP* Cardiac Arrest Hospital Prognosis, *RPRS* refractory post-resuscitation shock, *HIBI* hypoxic–ischaemic brain injury.

The cumulative incidence curves showed that, between CAHP deciles 5 and 10, the risk of RPRS as expressed by the sub-hazard ratio increased from one CAHP decile to the next (Fig. 4). In the tenth decile, the sub-hazard ratio for RPRS was 30.8 (95% CI 9.8–96.5; *p* < 0.0001) (ESM).

In contrast, the CAHP score did not seem to predict death from HIBI. The sub-hazard ratios for death from HIBI were not exponential between the fourth and ninth deciles and decreased for the tenth decile. The results did not differ in the sensitive analysis restricted to CA from cardiac cause and to initial shockable rhythm (ESM).

## Discussion

This study of two prospectively established population-based databases showed that CAHP score shows a discriminant power of this score to identify patients at risk of death. Indeed, higher CAHP scores at ICU admission after OHCA were associated with a higher risk of death due to RPRS. In contrast, the CAHP score did not predict death from HIBI.

The mortality rate in our study is consistent with the results of other studies done in Western countries^1,23,24^. Although guidelines issued in 2021 recommend collecting the reason for death as an Utstein variable^17,23,25–27^, such data remain sparse. One explanation may be the absence of a consensus about defining reasons for death. A 2019 retrospective study of 408 patients identified five reasons for death, namely, WLST warranted by HIBI, WLST warranted by co-morbidities, RPRS, recurrent CA, and respiratory failure^1^. In our study, HIBI was the most common reason for death, although the 45% proportion was lower than in earlier studies (65% and 73%)^1,24^, perhaps because these considered brain death a form of HIBI-related death, whereas we did not. Our finding that RPRS was the main cause of death in the first 3 days and HIBI later on is also in keeping with other data^24^.

The CAHP score was developed in France to predict outcomes of patients admitted to the ICU with ROSC after OHCA^11–13,29^. The predictive performance of the CAHP score has received robust external validation^12,13,28^. Scores fall into three groups, at low, moderate, and high risk for poor outcomes, respectively, with higher scores indicating worse outcomes. Poor outcome includes cerebral performance category (CPC) 3–4–5 without information of cause of death neither awakening before death. Instead, we distinguished CAHP score deciles to potentiate CAHP discrimination. In keeping with previous studies, we found that the mortality rate was 95% in patients with CAHP scores above 200.

The proportion of deaths due to RPRS was higher in patients with high CAHP scores in our competing-risks analysis. This finding is consistent with the reported association with post-resuscitation shock of three of the seven variables in the CAHP score, namely, low-flow time, arterial pH, and epinephrine dose^29^. Death by HIBI was not associated with the CAHP score. The seven variables are collected early, before or at ICU admission, whereas brain damage develops over time, with the possible participation of secondary insults^30,31^. Our competing-risks approach took into account the high early mortality from RPRS in patients with high CAHP scores.

Our findings suggest that the CAHP score may be useful to constitute uniform populations for future trials focus on post CA selecting the patients most likely to benefit from interventions aimed at preventing either RPRS or HIBI. Thus, patients with high CAHP scores at ICU admission may be unlikely to derive benefit from neuroprotective treatments such as TTM, given their high risk of early death due to RPRS. According to Sunde et al., patients at highest risk for poor outcomes should not be included in trials, as interventions may be beneficial only in low- and moderate-risk patients^32^. Using CAHP score deciles to conduct post hoc sub-group analyses of data from the recent trials of TTM might identify reasons for their divergent results. The efficacy of TTM has been found to vary according to the severity of post-CA syndrome, supporting the possibility that patient selection might improve outcomes^33^. In a population-based study, early coronary angiography was associated with higher survival in the low-risk CAHP-score group but not in the moderate- or high-risk groups^34^. An advantage of the CAHP score as an aid to treatment decisions is that the seven variables are available at ICU admission.

Several randomised controlled trials of interventions aimed at improving neurological outcomes are currently recruiting patients. To the best of our knowledge, none selects patients based on the risk of early death from RPRS, before neuroprotective interventions can show benefits. Our study indicates that the CAHP score might help to establish appropriate populations for evaluating neuroprotective treatments, excluding patients with a high risk of death from RPRS. A score specifically designed to predict neurological outcomes might also be useful. Moreover, neuroprotective treatment may be of no benefit in patients with minimal brain damage. Thus, the patient group most likely to benefit from TTM and other neuroprotective interventions may be the group at moderate risk.

Our study has several limitations. First, the design was retrospective, however, most of these data were collected prospectively. The medical resources and practices of the two recruiting centres in France may differ from those in other countries. Second, the reason for death was determined by at least two investigators, based on data available during the file review. For each reason for death, competition occurred with other reasons, but we tried to compensate for this by using a competing-risks regression models for reasons for death. Therefore, we don’t know the extent to which this competitive risk might contribute to the observed result that the CAHP score did not predict death from HIBI. The CAHP was designed for OHCA and we therefore confined our study to this sub-set, similar to most other studies of CA. The no-flow time, which is used to determine the CAHP score, can be difficult to estimate. Another variable used for CAHP-score determination is arterial pH, which may be modified by on-scene sodium bicarbonate administration^35^. We did not compare our results based on CAHP score to other existing scores using other prognostic variables. It would be interesting to develop a new score to answer the question of the best population to include in future CA trials. Given these limitations, our findings should be interpreted as preliminary and hypothesis-generating and should be tested in future studies.

## Conclusions

In conclusion, among reasons for death after OHCA, RPRS was more common in patients with high CAHP scores. This score may help to constitute uniform patient populations likely to benefit from interventions assessed in future randomised controlled trials. Further studies are warranted to assess this possibility.

## Supplementary Information

Supplementary Information.

## Data Availability

The datasets used and/or analysed during the current study are available from the corresponding author on reasonable request.

## Abbreviations

CA: Cardiac arrest
CAHP: Cardiac Arrest Hospital Prognosis
CI: Confidence interval
CPC: Cerebral performance category
CPR: Cardio pulmonary resuscitation
CT: Computerized tomography
ESM: Electronic supplementary material
HIBI: Hypoxic–ischemic brain injury
GCS: Glasgow Coma Scale
ICU: Intensive care unit
IQR: Interquartile-range
OHCA: Out-of-hospital cardiac arrest
ROSC: Return of spontaneous circulation
RPRS: Refractory post-resuscitation shock
SHR: Sub-hazard ratio
TTM: Targeted temperature management
WLST: Withdrawal of life-sustaining treatments
