# Supplementary material for: Association between the Cardiac Arrest Hospital Prognosis (CAHP) score and reason for death after successfully resuscitated cardiac arrest
Source: Sci Rep. 2023 Apr 13;13:6033. doi: 10.1038/s41598-023-33129-8 (PMC10102274; doi:10.1038/s41598-023-33129-8)
Supplement: Supplementary file 1 — Supplementary Information. [file 41598_2023_33129_MOESM1_ESM.docx]

**ELECTRONIC SUPPLEMENTARY MATERIAL**

**Evaluation of the Cardiac Arrest Hospital Prognosis (CAHP) Score for Predicting Reason for Death after Successfully Resuscitated Cardiac Arrest**

**ESM1: Management protocol for patients admitted after out-of-hospital cardiac arrest (OHCA)**

**ESM2: Neurological prognostication and criteria for withdrawal of life-sustaining treatments (WLST)**

**ESM3: Categorisation of reasons for death after out-of-hospital cardiac arrest (OHCA)**

**Table S1: Computing the Cardiac Arrest Hospital Prognosis score**

**Table S2: Cardiac Arrest Hospital Prognosis (CAHP) score deciles**

**Table S3: Reasons for death in each Cardiac Arrest Hospital Prognosis (CAHP) score decile**

**Table S4: Estimated sub-hazard ratios for each reason for death in each CAHP score decile, as assessed by competing-risks analysis**

**Table S5: Estimated sub-hazard ratios for each reason for death in each CAHP score decile, as assessed by competing-risks analysis in a sub group of patients with CA from cardiac cause n=857 (422 deaths)**

**Table S6: Estimated sub-hazard ratios for each reason for death in each CAHP score decile, as assessed by competing-risks analysis in a sub group of patients with an initial shockable rhythm n=761 (356 deaths)**

**ESM1: Management protocol for patients admitted after out-of-hospital cardiac arrest (OHCA)**

Immediately after OHCA, patients with no obvious non-cardiac cause of OHCA underwent coronary angiography followed, when appropriate, by a percutaneous coronary procedure. When the coronary angiography findings were inconclusive, brain and thoracic computed tomography-angiography were performed to look for reversible causes of cardiac arrest. Targeted temperature management was initiated immediately at ICU admission using external cooling by forced cold air during the first 24 hours to obtain a body temperature between 32°C and 36°C, as recommended. Rewarming to 36°C at a rate of 0.3°C/h was then started. Renal replacement therapy was initiated in patients with severe metabolic acidosis and/or life-threatening hyperkalaemia. The target mean arterial blood pressure (MAP) during the ICU stay was 65–75 mmHg. Post-resuscitation shock was defined as MAP<65 mmHg or systolic blood pressure<90 mmHg for more than six hours after the return of spontaneous circulation despite adequate fluid loading, requiring noradrenaline (norepinephrine), adrenaline (epinephrine), or dobutamine infusion. Clinical and non-convulsive seizures were treated with antiepileptic drugs (phenytoin, valproate, phenobarbital, levetiracetam). General anaesthesia was instituted using midazolam, propofol, or pentothal in patients with refractory status epilepticus. During the first 48 hours in the ICU, treatments were adjusted to maintain glucose control, normoxia, normocapnia, MAP at 65–70 mmHg, and haemoglobin above 7 g.dL^-1^.

Sedation was with combined midazolam and fentanyl before 2014 at one of the two participating centres (Cochin University Hospital, Paris), with a continuous infusion titrated to maintain a Richmond Agitation Sedation Scale (RASS) score of -5 (no response to voice or physical stimulation) until rewarming. The high frequency of delayed awakening with this regimen prompted a change to shorter-acting drugs, from 2015 onwards, with continuous combined propofol and remifentanil titrated to maintain the RASS score at -5 until rewarming. At the Versailles Hospital ICU, propofol was used alone starting in 2007 and neuromuscular blocking agents (NMBA) were given routinely until 2014; thereafter, shivering was appraised every 3 hours using the Bedside Shivering Assessment Scale score, the goal being no shivering (score of 0). Patients with scores above 0 received NMBA therapy. Sedation was interrupted after rewarming and the train-of-four response was assessed, with subsequent RASS monitoring being started only after clearance of the NMBA.

**ESM2: Neurological prognostication and criteria for withdrawal of life-sustaining treatments (WLST)**

After rewarming, the neurological prognosis was assessed daily in each patient by intensivists, until death or ICU discharge. In patients who were still comatose 72 hours after ROSC and after sedation discontinuation, a multimodal neuroprognostication protocol complying with international guidelines was applied, starting in 2007. The protocol included determining the Glasgow Coma Scale score, evaluating the pupillary and corneal reflexes, and performing an electroencephalogram (EEG) to rule out status epilepticus and to assess prognostic EEG patterns. Pupillary reflex and corneal reflexes were recorded every 3 hours by nurses and every 12 hours by physicians. Clinical or electrical status epilepticus was considered refractory when unimproved by two lines of major antiepileptic drugs (among phenytoin, fosphenytoin, valproate, phenobarbital, and levetiracetam). Standardised evaluation of N20 peaks on somato-sensory evoked potentials involved averaging the cortical EEG responses to repetitive electrical stimulation of the median nerve, which travelled to the contralateral post-central gyrus, producing a negative deﬂection on the recording about 20 ms after the stimulation. Technicians external to the ICU recorded bilateral absence of the N20 component with presence of P14 responses. A neurophysiologist consultant interpreted the recordings. Finally, serum neuron-specific enolase was assayed on day 3 after the cardiac arrest, and values above 80 ng/mL were considered to indicate a poor outcome. Patients without major predictors of poor outcome (i.e., with preserved N20 peaks and cranial reflexes and with a motor Glasgow Coma Scale score above 2) had their life-sustaining treatments continued and were further evaluated for causes of coma such as sepsis, residual sedation, intercurrent disease, or an underlying neurological disease. When no cause was found, a collegial decision to withhold and/or withdraw life-sustaining treatments was made based on all the available data. All patients who died after treatment-limitation decisions died in the ICU.

**ESM3: Categorisation of reasons for death after out-of-hospital cardiac arrest (OHCA)**

Death from hypoxic-ischaemic brain injury was defined as death after WLST warranted by a very poor neurological prognosis, based on the findings from the neuroprognostication protocol described above. In patients who died before being evaluated off sedation, evidence of severe neurological injury, such as severe cerebral oedema or cerebral herniation, was required to categorize the death as due to hypoxic-ischaemic brain injury.

Refractory post-resuscitation shock was progressive haemodynamic failure unresponsive to aggressive critical care leading to death either directly or after withdrawal of life-sustaining treatments (WLST) warranted by the haemodynamic failure. Thus, patients with stable haemodynamics including a stable mean arterial pressure of at least 65–70 mmHg when on full-dose vaso-active drug therapy were not considered to have refractory post-resuscitation shock.

Other causes of death in our study were brain death; recurrent cardiac arrest; WLST warranted by comorbidities; and shock developed secondarily due, for instance, to sepsis. Brain death was defined according to French standard criteria^1^. The diagnosis of BD was based on the French definition: clinical diagnosis of deep coma (Glasgow Coma Scale 3), loss of all brainstem reflexes, and the demonstration of apnea during a hypercapnia test with a rise in arterial partial pressure of carbon dioxide (PaCO2) after a 10-minute disconnection from a base value to ≥50 mmHg. Ancillary tests (e.g., cerebral CT angiograph or two isoelectric and unreactive electroencephalograms of 30 minutes duration, 4 hours apart) were used to confirm BD. Death by recurrent cardiac arrest was defined as recurrent cardiac arrest without subsequent return of spontaneous circulation, with or without advanced life-support measures such as the use of extracorporeal devices. WLST decisions warranted by comorbidities were taken in patients with a very poor expected quality of life in the event of survival, due to a pre-existing or newly diagnosed terminal illness or other serious illness such as advanced cancer or dementia. Secondary shock was defined as delayed haemodynamic failure due to a complication such as sepsis.

In patients with more than one plausible reason for death (e.g., refractory post-resuscitation shock and respiratory failure), an attempt was made to identify the primary reason of death (with or without WLST).

**Table S1: Computing the Cardiac Arrest Hospital Prognosis score**

| **Characteristic** | **Points** |
| --- | --- |
|  |  |
| **Age, years** | **1.1*(Age - 10)** |
| **Setting** | **0 if public place**  **24 if at home** |
| **Initial rhythm** | **0 if shockable**  **27 if non-shockable** |
| **No-flow time (min)** | **2.8*time** |
| **Low-flow time (min)** | **0.8*time** |
| **Arterial pH at ICU admission** | **585-77*pH** |
| **Total epinephrine dose during resuscitation (mg)** | **0 if 0 mg**  **27 if 1 or 2 mg** |
| **Total** | **Sum of sub-scores** |

**Table S2: Cardiac Arrest Hospital Prognosis (CAHP) score deciles**

| **Decile** | **Range (points)** | |
| --- | --- | --- |
| **q1** | **44.38** | **98.32** |
| **q2** | **98.45** | **123.52** |
| **q3** | **123.53** | **143.90** |
| **q4** | **144.10** | **158.12** |
| **q5** | **158.14** | **173.29** |
| **q6** | **173.31** | **187.55** |
| **q7** | **187.81** | **201.18** |
| **q8** | **201.24** | **217.95** |
| **q9** | **218.00** | **238.57** |
| **q10** | **238.61** | **362.22** |

q: quantile

**Table S3: Reasons for death in each Cardiac Arrest Hospital Prognosis (CAHP) score decile**

| **Decile (n of patients)** | **HIBI**  **N (%)** | **RPRS**  **N (%)** | **Other causes**  **N (%)** | **Survived to ICU discharge**  **N (%)** |
| --- | --- | --- | --- | --- |
| **q1 (N=155)** | 3 (1.9) | 7 (4.5) | 5 (3.2) | 140 (90) |
| **q2 (N=154)** | 5 (3.2) | 19 (12.3) | 15 (9.7) | 115 (74.7) |
| **q3 (N=154)** | 16 (10.3) | 28 (18.2) | 13 (8.4) | 97 (63) |
| **q4 (N=155)** | 9 (5.8) | 49 (31.6) | 18 (11.6) | 79 (51) |
| **q5 (N=154)** | 22 (14.3) | 59 (38.3) | 30 (19.5) | 43 (27.9) |
| **q6 (N=154)** | 30 (19.5) | 56 (36.4) | 28 (18.2) | 40 (26) |
| **q7 (N=155)** | 34 (21.9) | 72 (46.5) | 28 (18.1) | 21 (13.5) |
| **q8 (N=154)** | 43 (27.9) | 60 (39) | 36 (23.4) | 15 (9.7) |
| **q9 (N=154)** | 44 (28.6) | 68 (44.2) | 35 (22.7) | 7 (4.5) |
| **q10 (N=154)** | 85 (55.2) | 29 (18.9) | 39 (25.3) | 1 (0.6) |

Q: quantile; HIBI: hypoxic-ischaemic brain injury; RPRS: refractory post-resuscitation shock; ICU: intensive care unit

**Table S4: Estimated sub-hazard ratios for each reason for death in each CAHP score decile, as assessed by competing-risks analysis**

**a) CAHP deciles of patients who died from RPRS**


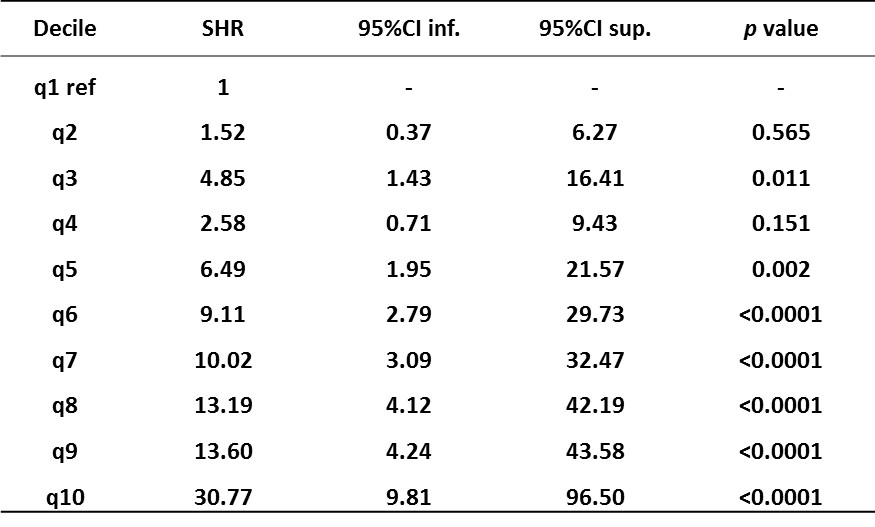


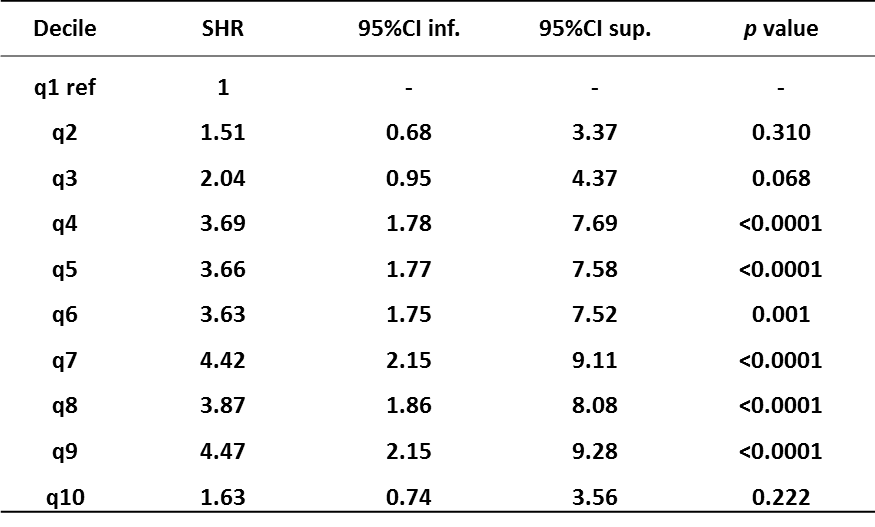
**b) CAHP deciles of patients who died from HIBI**

CAHP: Cardiac Arrest Hospital Prognosis; RPRS: refractory post-resuscitation shock; HIBI: hypoxic-ischaemic brain injury; SHR: sub-hazard ratio; 95%CI inf.: inferior boundary of the 95% confidence interval; 95%CI sup.: superior boundary of the 95% confidence interval

**Table S5: Estimated sub-hazard ratios for each reason for death in each CAHP score decile, as assessed by competing-risks analysis in a sub group of patients with CA from cardiac cause n=857 (422 deaths)**

**a-CAHP deciles of patients who died from RPRS**

| **Decile** | **SHR** | **95%CI inf.** | **95%CI sup.** | **p-value** |
| --- | --- | --- | --- | --- |
| CAHP score q1 ref | 1 | - | - | - |
| CAHP score q2 | 1.29 | 0.29 | 5.69 | 0.74 |
| CAHP score q3 | 4.33 | 1.24 | 15.15 | 0.02 |
| CAHP score q4 | 2.05 | 0.50 | 8.49 | 0.32 |
| CAHP score q5 | **8.35** | 2.44 | 20.57 | **0.001** |
| CAHP score q6 | **8.54** | 2.47 | 29.51 | **0.001** |
| CAHP score q7 | **12.94** | 3.84 | 43.63 | **<0.001** |
| CAHP score q8 | **16.84** | 5.08 | 55.86 | **<0.001** |
| CAHP score q9 | **12.33** | 3.51 | 43.30 | **<0.001** |
| CAHP score q10 | **30.33** | 9.34 | 98.45 | **<0.001** |

**b-CAHP deciles of patients who died from HIBI**

| **Decile** | **SHR** | **95%CI inf.** | **95%CI sup.** | **p-value** |
| --- | --- | --- | --- | --- |
| CAHP score q1 ref | 1 | - | - | - |
| CAHP score q2 | 1.54 | 0.59 | 4.04 | 0.38 |
| CAHP score q3 | **2.52** | 1.02 | 6.24 | **0.046** |
| CAHP score q4 | **4,46** | 1.82 | 10.91 | **0.001** |
| CAHP score q5 | **4.17** | 1.69 | 10.28 | **0.002** |
| CAHP score q6 | **4.21** | 1.73 | 10.24 | **0.002** |
| CAHP score q7 | **4.93** | 1.97 | 12.30 | **0.001** |
| CAHP score q8 | **4.37** | 1.73 | 11.01 | **0.002** |
| CAHP score q9 | **8.34** | 3.28 | 21.23 | **<0.001** |
| CAHP score q10 | 2.23 | 0.78 | 6.36 | 0.13 |

**Table S6 : Estimated sub-hazard ratios for each reason for death in each CAHP score decile, as assessed by competing-risks analysis in a sub group of patients with an initial shockable rhythm n=761 (356 deaths)**

**a-CAHP deciles of patients who died from RPRS**

| **Decile** | **SHR** | **95%CI inf.** | **95%CI sup.** | **p-value** |
| --- | --- | --- | --- | --- |
| CAHP score q1 ref | 1 | - | - | - |
| CAHP score q2 | 1.60 | 0.39 | 6.60 | 0.52 |
| CAHP score q3 | 4.47 | 1.29 | 15.47 | 0.02 |
| CAHP score q4 | 3.10 | 0.81 | 11.87 | 0.1 |
| CAHP score q5 | **9.31** | 2.69 | 32.24 | **<0.001** |
| CAHP score q6 | **9.96** | 2.87 | 34.54 | **<0.001** |
| CAHP score q7 | **12.91** | 3.80 | 43.92 | **<0.001** |
| CAHP score q8 | **18.38** | 5.39 | 62.69 | **<0.001** |
| CAHP score q9 | **14.45** | 3.86 | 54.05 | **<0.001** |
| CAHP score q10 | **31.43** | 8.97 | 110.12 | **<0.001** |

**b-CAHP deciles of patients who died from HIBI**

| **Decile** | **SHR** | **95%CI inf.** | **95%CI sup.** | **p-value** |
| --- | --- | --- | --- | --- |
| CAHP score q1 ref | 1 | - | - | - |
| CAHP score q2 | 1.52 | 0.64 | 3.62 | 0.34 |
| CAHP score q3 | 1.92 | 0.83 | 4.43 | 0.13 |
| CAHP score q4 | **3.34** | 1.45 | 7.67 | **0.005** |
| CAHP score q5 | **3.85** | 1.67 | 8.86 | **0.002** |
| CAHP score q6 | **3.95** | 1.76 | 8.89 | **0.001** |
| CAHP score q7 | **4.60** | 2.0 | 10.58 | **<0.001** |
| CAHP score q8 | **3.20** | 1.30 | 7.87 | **0.011** |
| CAHP score q9 | **5.53** | 2.15 | 14.25 | **<0.001** |
| CAHP score q10 | 2.0 | 0.65 | 6.24 | 0.23 |

**References**

1. Jousset, N., Gaudin, A., Mauillon, D., Penneau, M. & Rougé-Maillart, C. Organ donation in France: legislation, epidemiology and ethical comments. *Med Sci Law* 49, 191–199 (2009).
